# Comparative analysis of gene expression and metabolites in female and male *Cannabis sativa* flowers

**DOI:** 10.1016/j.isci.2026.114941

**Published:** 2026-02-07

**Authors:** Yang Chen, Guochao Qi, Jing Zhang, Zhigang Dai, Canhui Deng, Chaohua Cheng, Zemao Yang, Jiquan Chen, Xiaoyu Zhang, Siyuan Zhu, Qing Tang, Mingbao Luan, Ying Xu

**Affiliations:** 1 Institute of Bast Fiber Crops, Chinese Academy of Agricultural Sciences, Changsha 410205, China; 2 Daqing Branch of Heilongjiang Academy of Agriculture Science, Daqing 163319, China

**Keywords:** natural sciences, plant biochemistry, plant bioinformatics, plant Biology, plant physiology, plant development

## Abstract

*Cannabis sativa* L., a dioecious plant, exhibits sex-specific medicinal active ingredients. However, their causes remain unexplored. In this study, *C. sativa* flowers exhibited significant intersexual morphological differences, particularly in glandular trichomes. 1676 DEGs and 700 DAMs were identified. Among 149 genes related to sexual differentiation, 77 encode hormone-related products presumably involved in dioecy. Female flowers had cannabinoid levels 16.88 times those of males. Metabolomic sequencing identified 28 distinct terpenoids. We identified *CsDXPS1*, *CsLOX2*, *CsLOX1.5*, *CsAAE18*, *CsAAE6-1*, *CsAAE6-2*, *CsGGR,* and the metabolite mevalonate-5P; all except AAE6 were highly expressed in female flowers, regulating terpene and cannabinoid production. Seven glandular-trichome-related DEGs showed significant positive correlation with cannabinoid and terpene abundance; female-enriched *CsMYC4* (TPS activator) and *CsMYB49* (secretion enhancer) emerged as pivotal transcriptional regulators. These results provide a theoretical basis for the directional selection of female and male strains with high production and cultivation value.

## Highlights

• 1,676 DEGs and 700 DAMs are revealed in *Cannabis sativa* female and male flowers
• Female flowers show 16.88× cannabinoids and 28 distinct terpenoids versus males
• Mevalonate-5P and 8 DEGs increase cannabinoid/terpene biosynthesis in female flowers
• Seven trichome-DEGs and 112 TFs correlate with cannabinoid and terpene content

## Graphical abstract

**Figure:**
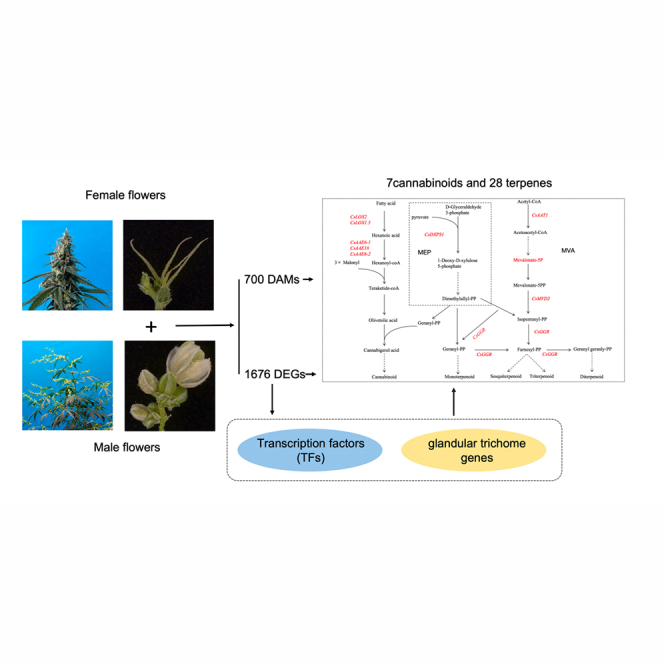

## Introduction

Dioecious plants evolved from hermaphroditic ancestors to avoid inbreeding depression and promote cross-pollination.^1^ Sexual specialization manifests as differences in morphology, function, biomass, and metabolic products between female and male plants.^2^ As such, a plant’s importance to consumers and economic value also tends to vary across sexes. For instance, female and male plants of the dioecious gymnosperm *Ginkgo biloba* L. have distinct uses^3^ that are reflected in morphological differences. The female plant's fruit contains various medicinal ingredients and nutritional value, whereas the medicinal value of the male plant is relatively low. Female plants are shorter than male plants at the same age, but have sturdier stems and more lateral branches. Female plants also have smaller leaves and shed leaves earlier than male plants.^4^ Male ginkgo has higher flavonoid content in roots, stems, and leaves than female ginkgo,^5^ whereas female plants have higher shikimic acid content in fallen leaves than male plants.^6^ In poplars, males have more metabolites of phenylpropane, polyketones, organic oxygen compounds, and lipids than females.^7^ Male kiwifruit strains are significantly higher in flavonoids, anthocyanins, total phenols, and free ubiquitin than female strains.^8^ However, female strains have higher free polyamine content than male strains.^9^^,^^10^^,^^11^ In papaya, testosterone is upregulated in male roots, whereas norgestrol is upregulated in female roots.^12^ In *Juniperus communis*, female shoots have significantly more germacrene than male shoots.^13^

Sexual differentiation in dioecious plants is genetically determined,^14^ with floral organ development regulated by MADS-box genes. In ginkgo flowers, *GbMADS16* varies across male flower development, but not across female flower development.^15^ In poplars, the MADS-box genes *APETALA3* (AP3) and *PISTILLATA* (PI) are key in stamen formation, and downregulation results in the production of female flowers.^16^ In *Salix viminalis*, *SvSAUR* is significantly associated with female gametophyte development and flower formation.^17^ In *Morella rubra*, chromosome 8 contains a female-specific region with genes unique to female plants (*MrASP2*, *MrCPS2*, *MrSAUR2*, and *MrFT2*).^18^ In red bayberry (*Myrica rubra*), the ethylene signaling pathway involves *MR1G019545.1* (ETR1), a gene associated with sex determination.^19^ In poplar and willow, *GAMYB* is male-flower-specific and important to pollen development.^20^ Female buds of *Actinidia chinensis* var. *chinensis* expressed Cdc5L at 2.22 times the level in male buds; this protein promotes fibroblast growth and could potentially be involved in the process of pollen formation.^8^ In female *Salix viminalis*, *SvTOGT1* and *SvHST* are significantly upregulated, affecting ovule development and the phenylpropyl and flavonoid metabolic pathways.^17^

*Cannabis sativa* is an annual, herbaceous, erect, and typically dioecious plant.^21^ *Cannabis sativa* has considerable economic value as an oil, fiber, and medicinal source. Female and male plants exhibit distinct morphology that influences their applications. The male plant of *C. sativa* has superior fiber quality to female plants, whereas female plants have more cannabinoids and other beneficial metabolites.^22^ The main organs responsible for the synthesis and storage of cannabinoids and terpenes are the secretory glandular trichomes in *C. sativa*; unsurprisingly, these structures have a higher distribution on the bracts of female flowers than of male flowers.^23^^,^^24^ The cannabinoid content varies in different organs, with the content in inflorescences being higher than that in leaves.^25^ Furthermore, the cannabinoid content in cannabis is influenced by genetic factors,^25^ and by environmental and cultivation conditions,^26^ such as stress conditions,^27^ mineral nutrition,^28^^,^^29^ and planting density.^30^ The sex ratio of *C. sativa* is influenced by hormones such as GA3, 6-BA, and IAA.^31^^,^^32^ Transcriptome sequencing identified approximately 200 genes that participate in male organ development (anthers and pollen) through hormone signal transduction.^33^

Although some studies have investigated intersexual differences in secretory metabolites and gene expression of *C. sativa*, combined transcriptomic and metabolomic analyses of these differences have not been performed. Therefore, this study conducted a joint analysis of female and male *C. sativa* flowers to quantify intersexual differences in metabolites and gene expression. This study provides a foundation for the cultivation, selection, and function-directed breeding of *C. sativa* female and male plants.

## Results

### Morphological difference between female and male flowers

**Figure 1:**
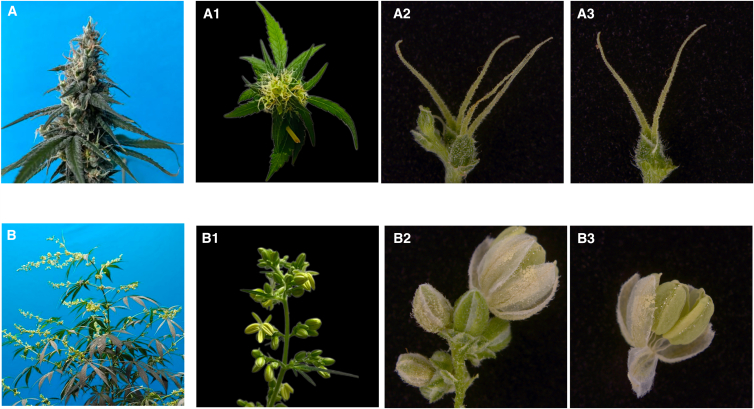
Morphological differences between female and male flowers (A) Female flower inflorescence: A1-3, multiple female flowers and single female flowers. (B) Male flower inflorescence: B1-3, multiple male flowers and single male flowers.

Female and male *C. sativa* flowers differed visually (Figure 1).^34^ Female flowers (Figure 1A) were spike-like, green, with dense inflorescences and without obvious floral organs (Figure 1A1-2). The pistil comprised a thin, transparent sepal envelope, without a petalous stalk. The ovary was composed of a bilocular carpel. The stigma was filamentous and bilobated. Both stigma and sepal surfaces were covered with glandular hairs (Figure 1A3).

Male flowers were compound racemes with loose inflorescences (Figure 1B), distinct floral organs (Figure 1B1-2), stalks, five yellowish-green sepals, and five stamens (Figure 1B3). Under an inverted microscope, female flowers had higher secretory glandular hair density than male flowers. Any glandular hairs on male flowers were non-secretory.

### Differential gene expression of female and male flowers

**Figure 2:**
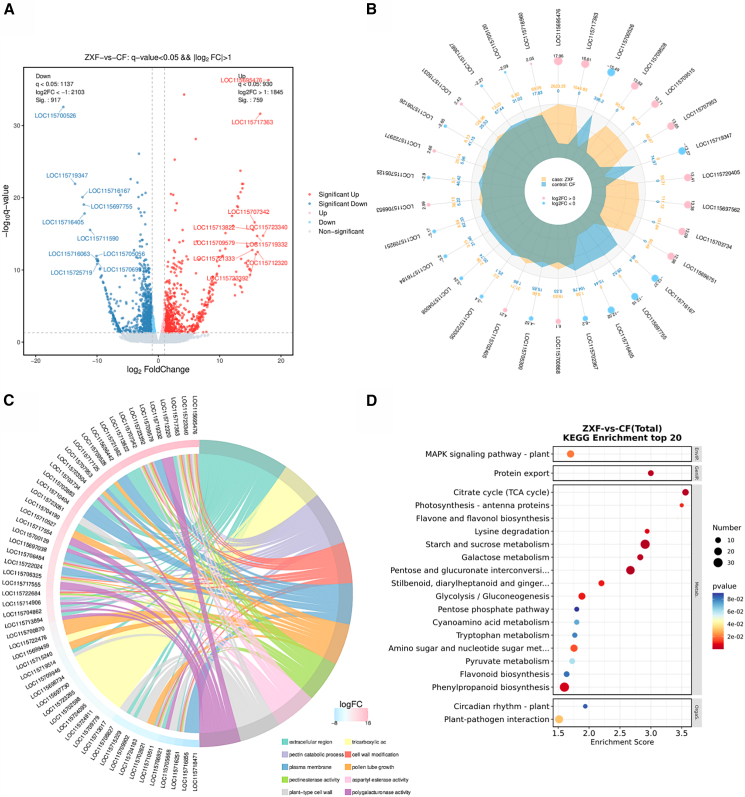
Analysis of differentially expressed genes in female and male *Cannabis sativa* L (A) Volcano map of differential gene expression. (B) Differential gene radar map of the top 30 significantly up- and downregulated genes. (C) Chord map of the top 10 terms after GO enrichment analysis. (D) Bubble map of the top 20 KEGG pathways in XF (male) and CF (female).

Transcriptome sequencing with high throughput resulted in the identification of 19,203 genes, from which we screened out 1676 differentially expressed genes (DEGs) (fold-change >2, Q-value ≤0.05). Of these DEGs, 759 were highly expressed in male flowers, and 917 were highly expressed in female flowers (Figure 2A; Table S1), whereas 112 genes were not expressed in male flowers and 118 were not expressed in female flowers. The 30 most significant DEGs included four genes not expressed in female flowers and nine genes not expressed in male flowers (Figure 2B).

Gene Ontology (GO) analysis was performed on 1196 DEGs (Figure S1), revealing their enrichment in 45 GO_classify2. The top three categories of “cellular component” were cell (920 DEGs), cell part (919 DEGs), and cellular process (681 DEGs). The “biological processes” domain contained the largest number of DEGs, with the top 10 most significant classes (Figure 2C) including pectinesterase activity (GO: 0030599), tricarboxylic acid cycle (GO: 0006099), pectin catabolic process (GO: 0045490), and extracellular region (GO: 0005576). We identified 55 genes; 27 genes were not expressed in female flowers, whereas two (*LOC115718471* and *LOC115716855*) were not expressed in male flowers (Table S2).

Next, the Kyoto Encyclopedia of Genes and Genomes (KEGG) analysis of 537 DEGs (Figures 2D and S2) revealed enrichment in signal transduction (36 DEGs), biosynthesis of secondary metabolites (38 DEGs), and carbohydrate metabolism (111 DEGs). Secondary metabolite synthesis included the phenylpropane biosynthesis pathway (ko00940) (31 DEGs), flavone and flavonol biosynthesis (ko00944) (3 DEGs), flavonoid biosynthesis (ko00941) (10 DEGs), stilbenoid, diarylheptanoid, and gingerol biosynthesis (ko00945) (11 DEGs), and the MAPK signaling pathway (ko04016) (17 DEGs). In summary, female and male flowers demonstrated significant differences in gene expression. These genes regulate the differential secretion of metabolites in male and female plants and influence their environmental adaptability through signal transduction and environmental responses.

### Identification of sex-determination genes

We identified 149 DEGs that could be associated with the sexual differentiation of female and male flowers; 72 were related to flower organs (Table S3), whereas 50 were associated with pollen growth, development, maturation, and regulation. In male flowers, 42 DEGs were upregulated. 13 DEGs were not expressed in female flowers, and the other 29 DEGs were downregulated. The remaining eight DEGs were highly expressed in female flowers. One DEG (*LOC115709438*) was associated with stamen development. Additionally, 21 DEGs were related to embryo development: 15 were highly expressed in female flowers, four were inhibited, and two were not expressed. In male flowers, six were highly expressed, 13 were inhibited, and two were not expressed. Two DEGs (*LOC115700786* and *LOC115708058*) were associated with embryo sac and pollen development. *SPH5* (*LOC115721982*) was responsible for rejecting self-pollination and was highly expressed only in male flowers (Table S2).

We also identified 77 DEGs responsible for sexual differentiation, specifically participating in hormone biosynthesis, signal transduction, catabolism, and regulation (Table S4). Of these, 31 were highly expressed in male flowers and suppressed in female flowers, whereas the reverse was true for the remaining 46. Among 77 DEGs, 46 DEGs were associated with auxin, 9 were related to gibberellin, 23 were related to ethylene, and 14 were related to cytokinin. LOC115702452 was associated with three hormones other than CTK, and 12 DEGs were associated with two hormones.

### Differential metabolites accumulate in female and male *C. sativa* flowers

**Figure 3:**
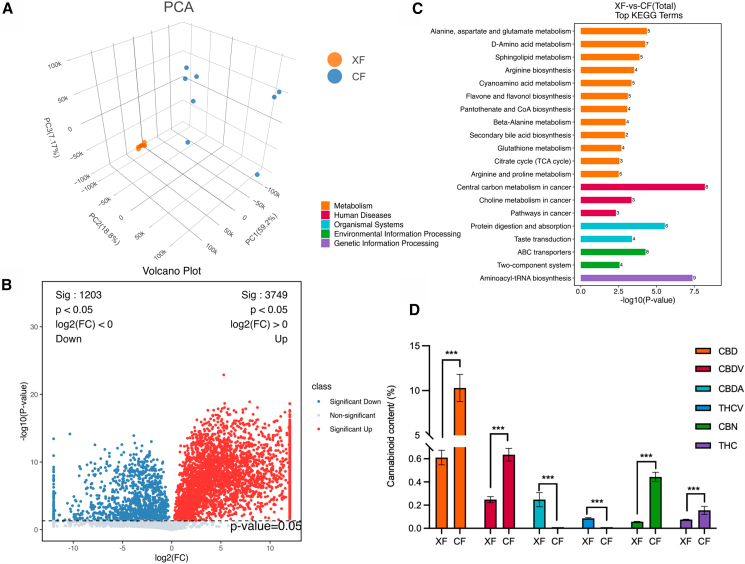
Differentially accumulated metabolites (DAMs) in female and male *Cannabis sativa* L (A) Principal component analysis (PCA) of metabolites. (B) Volcano plot of DAMs. (C) Histogram of the top 20 most enriched KEGG pathways. (D) Cannabinoid content in female and male *C. sativa.* “∗∗∗” indicates significant differences at the *p* < 0.001 levels. Data are represented as mean ± SEM.

The LC-MS metabolomics analysis of female and male flowers identified 18,678 metabolites. All samples fell within the range of the 95% confidence level after principal component analysis (PCA, Figure 3A), indicating that the metabolomic data were reliable. The first three components explained 61.5%, 7.08%, and 5.19% of total variance, respectively. Further analysis revealed 700 differentially accumulated metabolites (DAMs) between female and male flowers (Table S5); 480 were more abundant in female flowers than in male flowers, and 220 were less abundant. We classified 588 (out of 700, excluding 112 unclassified) DAMs into 63 categories. Fatty acids were the largest category (125 DAMs) (Table S6).

Functional analysis of DAMs showed that 135 metabolites were enriched in 131 pathways (Table S7). The top 20 metabolic pathways were related to metabolism (12 pathways), human diseases (three), organismal systems (two), environmental information processing (two), and genetic information processing (one) (Figure 3C). Notably, aminoacyl-tRNA biosynthesis and central carbon metabolism pathways in cancer had the highest number of enriched metabolites (nine and eight, respectively). Thus, DAMs are linked to human health, specifically cancer, digestion, and metabolism.

### Intersexual differences in cannabinoid and terpene content

The main metabolites found in hemp are cannabinoids, terpenes, and alkaloids. Cannabinoids were particularly abundant (a quarter of all metabolites). After measuring six common cannabinoids (cannabidiol, CBD; cannabidivarin, CBDV; cannabidiolic acid, CBDA; tetrahydrocannabivarin, THCV; tetrahydrocannabinol, THC; cannabinol, CBN), we observed that the contents of CBD, CBDV, CBN, and THC were significantly higher in female flowers than in male flowers (Figure 3D). Additionally, CBDA and THCV contents were significantly higher in male flowers than in female flowers (Figure 3D). Finally, CBD was highest in female flowers, being 16.88 times the CBD content in male flowers.

The second most abundant metabolite after cannabinoids was terpenes, the source of cannabis’s unique aroma. Their antioxidant and anti-inflammatory effects synergize and complement cannabinoids.^35^ Metabolomic sequencing uncovered 28 differentially abundant terpenoid compounds in female and male flowers (Table 1). Fourteen were more abundant in female flowers than in male flowers, whereas the other 14 were richer in male flowers. Sesquiterpenoids (16 subtypes) were the most abundant terpenoids, whereas piperoic acid and beta-farnesene contents were high in both female and male flowers; piperoic acid was even more elevated in female flowers.

**Table 1:** Differentially abundant terpenes in female and male *Cannabis sativa* L. flowers

Metabolites	Subclass	Average (XF)	Average (CF)	log2FoldChange
3D,7D,11D-Phytanic acid	Diterpenoids	11778116.79	6697.69	10.78016
Annosquamosin B	Diterpenoids	9665044.76	850444.91	3.50649
Sagittariol	Diterpenoids	6072231.37	552448.64	3.45831
Sugiol	Diterpenoids	70981.58	2280254.07	−5.00561
3L,7D,11D-Phytanic acid	Diterpenoids	7090.82	1532524.10	−7.75574
3-(2-Hydroxy-4-methylphenyl)-2- butanone	Monoterpenoids	25918.00	4676748.59	−7.49541
Dihydrocarvone	Monoterpenoids	2765855.28	820167.75	1.75373
Piperoic acid	Sesquiterpenoids	269067159.50	595832443.30	−1.14694
beta-Farnesene	Sesquiterpenoids	108091083.80	45858409.11	1.23699
Armillarin	Sesquiterpenoids	23690640.95	44382489.05	−0.90567
(4E,9a)-9-(3-Methyl-2E- pentenoyloxy)-4,10(14)-oplopadien- 3-one	Sesquiterpenoids	279180.52	9175276.98	−5.03848
11′-Carboxy-alpha-tocotrienol	Sesquiterpenoids	5880223.49	1834.88	11.64597
9alpha-(3-Methyl-2E-pentenoyloxy)-4S-hydroxy-10(14)-oplopen-3-one	Sesquiterpenoids	379231.79	8152925.49	−4.42617
6-Angeloylfuranofukinol	Sesquiterpenoids	219330.34	3754380.89	−4.09740
Gossyvertin	Sesquiterpenoids	5917646.07	1438623.07	2.04033
Tricyclodehydroisohumulone	Sesquiterpenoids	1106155.31	4403164.66	−1.99299
trans,trans-Farnesol	Sesquiterpenoids	2902010.87	77347.94	5.22955
Gossypol	Sesquiterpenoids	3241810.37	704375.12	2.20238
4,8-Diacetyl-T2-tetrol	Sesquiterpenoids	208360.71	2506415.29	−3.58847
Acoragermacrone	Sesquiterpenoids	868485.63	2853159.20	−1.71599
Zedoarondiol	Sesquiterpenoids	2419854.00	528246.51	2.19564
2-Angeloyl-9-(3-methyl-2E- pentenoyl)-2b,9a-dihydroxy- 4Z,10(14)-oplopadien-3-one	Sesquiterpenoids	2206702.18	460309.23	2.26122
Armillyl orsellinate	Sesquiterpenoids	8547.65	1672261.93	−7.61206
Fasciculol C	Triterpenoids	2914511.77	2956.10	9.94534
Ganoderic acid H	Triterpenoids	3198766.04	24.18	17.01342
Reticulataxanthin	Triterpenoids	70771.22	2270579.68	−5.00375
Neochlorogenin	Triterpenoids	1881862.52	76.22	14.59156
Protobassic acid	Triterpenoids	1780406.11	3829.12	8.86098

### Integrative analysis of metabolomics and transcriptomics

To further reveal the differences between female and male flowers, an integrated analysis of the transcriptome and metabolome of female and male flowers was conducted. We mapped 358 DEGs and 135 DAMs to 53 KEGG pathways. Citric acid cycle, ABC transporters, and phenylpropane biosynthesis were the most differentially enriched pathways in female and male flowers (Figure S3). All 13 of the DEGs enriching the citric acid cycle were highly expressed in male flowers. Additionally, two DAMs (malic acid and citric acid) were more highly accumulated in male flowers, whereas isocitrate was more highly accumulated in female flowers. The growth hormone gibberellin A15 was extremely abundant in female flowers. Among the four DEGs involved in ABC transporters, three (*ABCB11*, *ABCB1,* and *ABCC10*) were upregulated in female flowers, and one (*ABCB8*) was upregulated in male flowers.^36^ Eight DAMs were more highly accumulated in male flowers.

Of the 31 DEGs associated with phenylpropanoid biosynthesis, 23 were highly expressed in female flowers and 8 in male flowers. We also identified two DAMs (L-phenylalanine and spermidine) that were highly expressed in male flowers. 31 DEGs and two DAMs led to the difference in Phenylpropanoid metabolism between males and females. Phenylpropanoid metabolism is closely related to the synthesis of numerous medicinally active components. Almost all natural pharmacological ingredients containing a phenylpropanoid skeleton are synthesized directly or indirectly through the phenylpropanoid metabolic pathway, such as flavonoids, terpenoids, and phenolics. This could be one of the reasons for the differences in terpene and cannabinoid production between female and male flowers.

### Transcription factors related to cannabinoids and terpenes

**Figure 4:**
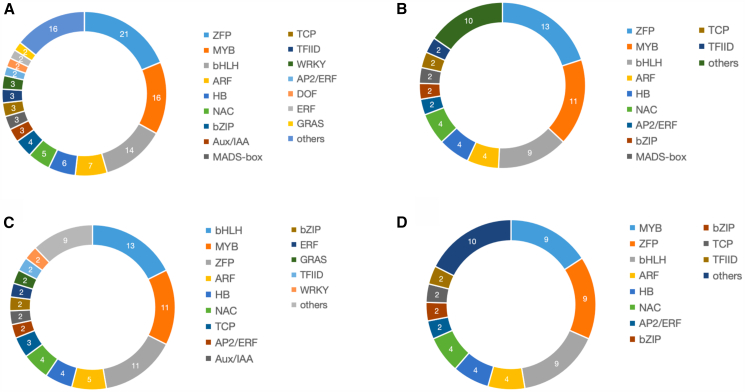
Transcription factors (TFs) related to cannabinoids and terpenes (A) Related to either cannabinoids or terpenes. (B) Related to cannabinoids only. (C) Related to terpenes only. (D) Related to both terpenes and cannabinoids.

We identified 112 differentially expressed TFs (Table S8); 84 were upregulated in female flowers, and 28 were upregulated in male flowers. The ZFP family was the most well-represented, with 21 genes. The MYB (16 genes), the basic-helix-loop-helix (bHLH) (14), and ARF (7) families (Figure 4A) follow closely. Of the 65 TFs (22 families) associated with cannabinoids (R ≥ 0.8 and Q-value ≤0.05), ZFP was again the most well-represented (13 DEGs), followed by MYB (11) and bHLH (9) (Figure 4B). Of the 74 TFs (24 families) associated with 26 terpenes (R ≥ 0.8, Q-value ≤0.05), the most well-represented were bHLH (13 DEGs), MYB (11), and ZFP (11) (Figure 4C). Finally, 58 TFs were associated with both cannabinoids and terpenes (Figure 4D).

### The differences in cannabinoid and terpene synthesis in female and male *C. sativa* flowers

**Figure 5:**
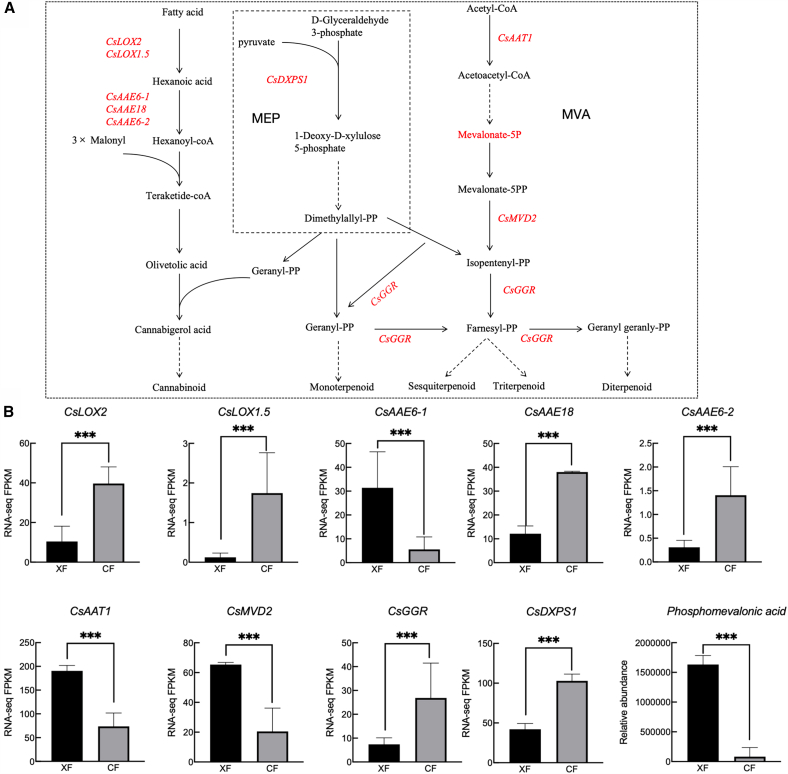
Genes and metabolites of terpene and cannabinoid synthesis pathways (A) Schematic of terpene and cannabinoid synthesis pathways. (B) DEGs and DAMs involved in these pathways.“∗∗∗” indicates significant differences at the *p* < 0.001 levels. Data are represented as mean ± SEM.

Cannabinoid and terpene synthesis share the 2-C-methyl-D-erythritol-4-phosphate (MEP) pathway (Figure 5A). Cannabinoid precursors are geranyl diphosphate (GPP) and olivetolic acid (OLA); the former is produced via the MEP pathway and the latter via polyketone synthesis. Lipoxygenase (LOX) and acyl-activating enzymes (AAEs) are the key enzymes during polyketide synthesis. The metabolic precursors of terpenes are produced via the MEP or mevalonate (MVA) pathways. The first key enzyme of the MEP pathway is 1-deoxy-D-xylulose-5-phosphate synthase (DXPS). Isopentenyldiphosphate (IPP) and dimethylallyldiphosphate (DMAPP) are condensed to GPP by geranyl diphosphate synthase (GPPS), an isoprene diphosphate synthase (IDS) that determines terpenoid type. Terpene dehydrogenases (TPS) then act on GPP, FPP, and GGPP to form monoterpenes, sesquiterpenes, and diterpenes, respectively. The MVA pathway intermediate mevalonate-5P can be converted into various isoprenoids that play important roles in cell structure and function.^37^

We identified *CsDXPS1* (*LOC115722576*), *CsLOX2* (*LOC115720530*), *CsLOX1.5* (LOC115724062), *CsAAE18* (*LOC115722340*), *CsAAE6-1* (*LOC115695955*), *CsAAE6-2* (*LOC115724480*), *CsGGR* (*LOC115725388*), *CsAAT* (*LOC115699135),* and *CsMVD*2 (*LOC115705753*) involved in the synthesis pathways of terpenes and cannabinoids (Figure 5A). Except for *CsAAE6-1*, *CsAAT,* and *CsMVD2*, all the identified genes were highly expressed in female flowers (Figure 5B). *CsGGR* (*LOC115725388*) was associated with multiple pathways, including terpenoid backbone biosynthesis, sesquiterpenoid and triterpenoid biosynthesis, monoterpenoid biosynthesis, diterpenoid biosynthesis, and indole diterpene alkaloid biosynthesis (Figure S4). We also discovered that one DAM (Mevalonate-5P) exhibited elevated accumulation in male flowers, affecting terpene synthesis. These DEGs and DAMs are the likely intersexual variation sources in the 28 terpenes and cannabinoids we identified.

## Discussion

### Determinants of sex expression and consequences for medicinal properties in male and female plants

Auxin, gibberellin, cytokinin, and ethylene all play critical regulatory roles in plant sex differentiation. Auxin primarily functions in plant cell elongation and division, as well as the promotion of female traits.^38^ Similarly, ethylene and cytokinin promote female floral bud differentiation.^39^^,^^40^ In contrast, gibberellin generally inhibits flowering and promotes male differentiation^41^^,^^42^ (although they appear to trigger pistil development in corn^43^). Given the morphological differences between female and male *C. sativa* flowers, we uncovered DEGs related to sex differentiation, specifically 77 genes associated with major plant hormones (auxin, gibberellin, cytokinin, ethylene) and their biosynthesis pathways.^44^ First, the citric acid cycle provides precursor substances and energy for plant hormone biosynthesis.^45^ We also observed ABC transporter upregulation, crucial for development processes (e.g., gametogenesis, germination, and organ formation), given their function in transporting essential products throughout the plant.^46^ ABCB1 mediates auxin transport. Meanwhile, ABCC10 is directly involved in the transport of the auxin precursor indole-3-butyric acid (IBA). Both ABCB1 and ABCC10 play crucial roles in plant growth and development.^47^^,^^48^These hormones and genes may contribute to the sex differences between female and male *C. sativa* flowers.

Cannabinoids and terpenes are the most important components in medicinal and industrial *C. sativa.* In this study, we confirmed previous research^22^^,^^23^^,^^24^ and demonstrated that female and male *C. sativa* differ significantly in cannabinoid and terpene content. Particularly, female plants have 16.88 times the amount of CBD as male plants. Because CBD is an important medicinal ingredient, along with piperoic acid and sugiol, the higher concentrations in female plants indicate stronger medicinal applications than in male plants, this is consistent with the results of previous studies.^24^ Therefore, *C. sativa* cultivation should target female-only or female-dominant varieties, if the primary goal of cultivation is to maximize the plant’s medicinal properties, particularly CBD production. Furthermore, we found 9 DEGs and 1 DAM in the synthesis of cannabinoids and terpenes (Figure 5B). They specifically promote and inhibit the cannabinoid and terpene synthesis, resulting in differences in medicinal functions between male and female *C. sativa*, providing reference genes for the targeted breeding and selection of medicinal cannabis.

### Factors underlying cannabinoids and terpenes differences between male and female C. sativa

**Figure 6:**
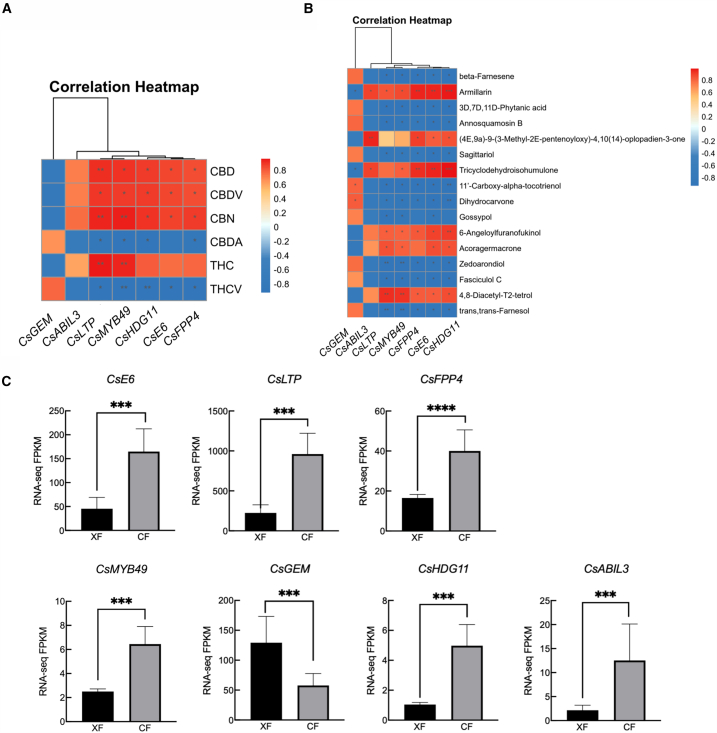
Heat maps of glandular hair genes associated with terpenes and cannabinoids (A) Correlation between glandular hair genes and cannabinoids. (B) Correlation between glandular hair genes and terpenes. (C) Expression of seven glandular trichome genes in female and male flowers. “∗∗∗” indicates significant differences at the *p* < 0.001 levels. Data are represented as mean ± SEM.

Glandular trichomes participate in plant defense responses, as well as the synthesis and storage of secondary metabolites^49^ such as cannabinoids and terpenes in female *C. sativa* flowers.^50^ In support of this function, female flowers had the highest density of secretory glandular trichomes (Figure 1). Two TF families are particularly important players in trichrome and plant epidermal hair development: HD-ZIP IV and MYB. In *Arabidopsis*, *HDG11* is involved in trichome development, whereas *HDG11* and *HDG12* are associated with the branching of glandular trichomes.^51^ Additionally, *AtMYB106* is associated with trichome development and positively regulates plant epidermal hair formation.^52^ Similarly, in poplar, overexpressing the TF *PtaMYB186* significantly increased epidermal hair density on leaves.^53^ We also found seven DEGs related to glandular trichomes (Figure 6C), and all except *CsGEM* (*LOC115713063*) were highly expressed in female flowers. Five DEGs were significantly associated with cannabinoids (Figure 6A) and seven with terpenes (Figure 6B). *CsLTP* (*LOC115698127*) and *CsMYB49*(*LOC115708954*)^54^ were linked to 6 cannabinoids and 15 terpenes. *CsHDG11* (*LOC115714770*) was correlated with 5 cannabinoids and 15 terpenes, including CBD. We also found that secretory glandular trichomes were more common in female flowers than in male plants.^23^^,^^24^ Based on their functions in other plants, these genes likely regulate the development of glandular trichomes in female plants, thereby indirectly affecting the variation in cannabinoid and terpene content between sexes, making female plants more suitable for medicinal use.

In *Arabidopsis*, *AtMYC2* directly binds to and activates sesquiterpene synthase genes *AtTPS11* and *AtTPS21* through synergistic gibberellin and jasmonic acid regulation, leading to sesquiterpene release.^55^ In *Artemisia annua*, *AaMYB17* is a positive GST initiation regulator in glandular secretory trichomes and increases artemisinin content.^56^ In tomatoes, *SlMYB75* enhances the production of volatile compounds, including terpenes, specifically by acting on the promoters of LOXC, AADC2, and TPS genes.^57^ In spearmint, MsMYB inhibits GPPS activity via its promoter region and limits monoterpene accumulation.^58^ Finally, co-expression analysis of terpene metabolites and DEGs revealed that bHLH and MYB TFs control tea aroma by regulating terpene volatiles via the MVA and MEP pathways.^59^ We found that bHLH and MYB families are significantly correlated with the synthesis of cannabinoids and terpenoids (Figures 6A and 6B). One notable bHLH member we found was the MYC4 (*LOC115708126*), which regulates jasmonic acid synthesis and terpene synthase (TPS)^60^. This function is in line with research in other plants, demonstrating that MYC2 is a significant regulator of jasmonic acid signaling. The main MYB member we found was *CsMYB49* (*LOC115708954*), highly expressed in female flowers and associated with both terpenes and cannabinoids. Taken together, available data reflect the clear importance of TFs in regulating plant terpene and cannabinoid secretion.

### Limitations of the study

*Cannabis sativa* has considerable economic value as an oil, fiber, and medicinal source. Female and male plants exhibit distinct morphology that influences their applications. To elucidate the sex-biased genes and metabolic disparities underlying these differences, we employed integrated transcriptomic and metabolomic profiling of flowers. The principal chemotypic divergence resides in cannabinoids and terpenoids: we found 9 DEGs and 1 DAM in the synthesis of cannabinoids and terpenes. We infer that these genes indirectly modulate sexual dimorphism in cannabinoid and terpene content; however, the precise regulatory mechanisms remain to be experimentally dissected. We also found that two TF families are particularly important players in trichome and plant epidermal hair development. One notable bHLH member we found was the MYC4 (LOC115708126), and the main MYB member we found was CsMYB49 (LOC115708954). Available data reflect the clear importance of TFs in regulating plant terpene and cannabinoid secretion, yet the molecular details by which these TFs govern glandular-trichome secretion require future investigation. Finally, *C. sativa* also offers oil- and fiber-utilization potential; the present study focused exclusively on its medicinal attributes, while oil- and fiber-related traits will be explored in future research.

## Resource availability

### Lead contact

Requests for further information and resources should be directed to and will be fulfilled by the lead contact, Ying Xu, E-mail: xuying0420@163.com.

### Materials availability

This study did not generate new, unique reagents.

### Data and code availability

• Data: The raw sequence data reported in this article have been deposited in the Genome Sequence Archive (Genomics, Proteomics & Bioinformatics 2025) in the National Genomics Data Center (Nucleic Acids Res 2025), China National Center for Bioinformation/Beijing Institute of Genomics, Chinese Academy of Sciences (GSA: CRA036554) that are publicly accessible at https://ngdc.cncb.ac.cn/gsa/search?searchTerm=CRA036554. Accession numbers are listed in the key resources table.
• Code: This article does not report original code.
• Any additional information required to reanalyze the data reported in this article are available from the lead contact upon request.

## Acknowledgments

This study was financially supported by the Germplasm Resources Protection Project and the Central Public-interest Scientific Institution Basal Research Fund (1610242024015). We sincerely thank Editage (www.editage.cn) for English language editing.

## Author contributions

Y.C.: conceptualization, methodology, investigation, data curation, formal analysis, and writing - original draft. G.Q.: investigation, data curation, and formal analysis. Jing Zhang: investigation and data curation. Z.D.: review and editing. C.D.: investigation and data curation. C.C.: review and editing. Z.Y.: review and editing. J.C.: visualization. X.Z.: review and editing. S.Z.: review and editing. Q.T.: methodology and supervision. M.L.: methodology and supervision. Y.X.: conceptualization, methodology, formal analysis, and funding acquisition.

## Declaration of interests

The authors declare there is no conflict of interest in this article.

## STAR★Methods

### Key resources table

**Table:** 

REAGENT or RESOURCE	SOURCE	IDENTIFIER
**Biological samples**		

Long 6	the National Germplasm MId-term GenBank of Bast Fiber Crops,in the Institute of Bast Fiber Crops of the Chinese Academy of Agriculture Sciences	N/A

**Chemicals, peptides, and recombinant proteins**		

CBD standards	Sigma Aldrich (St. Louis, USA)	C-045
CBDA standards	Sigma Aldrich (St. Louis, USA)	C-144
CBDV standards	Sigma Aldrich (St. Louis, USA)	C-199
THC standards	Sigma Aldrich (St. Louis, USA)	T-005
THCV standards	Sigma Aldrich (St. Louis, USA)	T-111
CBN standards	Sigma Aldrich (St. Louis, USA)	C-093

**Deposited data**		

Transcriptome sequencing data	This article	https://ngdc.cncb.ac.cn/gsa/search?searchTerm=CRA036554

**Software and algorithms**		

Progenesis QI version 3.0	Nonlinear Dynamics, Newcastle, UK	https://www.nonlinear.com/progenesis/qi/v3.0/download/
DESeq2 V1.22.2	Ref. 68	https://bioconductor.org/packages/release/bioc/html/DESeq2.html
R software V 3.2.0	R Development Core Team	https://cran.r-project.org/

### Experimental model and study participant details

The industrial *C. sativa* variety Long 6 was provided by the National Germplasm Mid-term GenBank of Bast Fiber Crops in the Institute of Bast Fiber Crops of the Chinese Academy of Agriculture Sciences. This particular variety represents a medicinal-grade industrial hemp with characteristically high CBD content. For propagation in artificial climate-controlled room, lateral branches (approximately 12 cm) of female and male plants were cultivated in a plastic basin (diameter = 32 cm) and cultured in an artificial culture chamber maintained at 50–60% relative humidity and 25 ± 2 °C. Light conditions were 33,000 lux, with 18 h of light and 6 h of darkness. During the growth period, cuttings were watered once every 3 days to keep the soil moist. The seedling matrix (pH 5.5–7.0, organic matter content ≧20%) was acquired from Hunan Xianghui Agricultural Science and Technology Development. Detailed methods followed a published protocol.^61^ Compound fertilizer, with a composition of 30 g Pot-1, 30% N, 10% K, and 10% P, was applied at 14 days after the emergence of the first new leaf. After 4 weeks of vegetative growth, female and male plants of approximately 60 cm in height were selected and transferred to a closed artificial climate chamber. The light-dark cycle was set to 12 h of light and 12 h of darkness, whereas the temperature and humidity remained unchanged. After 28 days of cultivation, the light-dark cycle was adjusted to 8 h of light and 16 h of darkness to induce reproductive growth and flowering. Female and male plants entered the peak bloom period after 26 days and 35 days, respectively. All samples were obtained during the peak bloom period to ensure biological consistency. The flower sampling site was approximately 2 cm from the apex. Flowers were photographed before sample collection for cannabinoid, transcriptome, and metabolome analyses.

For cannabinoid determination, female plants were sampled at 5 cm from the top of the flower, and male plants at 3–8 cm from the top (5 g per sample); sampling was repeated three times. For transcriptome and metabolome analysis, a separate 1 g of plant material (from the same positions) was obtained. Samples were put into a 5 mL freezer tube and subsequently immersed in liquid nitrogen for freezing. Transcriptome analysis was performed in triplicate per sex for a total of six samples. Metabolomic analysis was repeated eight times per sex for 16 samples.

### Method details

#### Cannabinoid extraction and analysis

Female and male flower samples were collected, dried at 85 °C until they reached a constant weight, and ground before methanol (4 mL) was added. The mixture was processed using ultrasonic extraction for 10 min and then stood for 1 h. Next, the samples underwent centrifugation at 4000 r/min for 5 min, after which the supernatant was transferred to a 10 mL volumetric flask for calibration. Using a syringe, 1 mL of supernatant was filtered through a 0.45 μm microporous membrane, transferred to a 2 mL liquid phase sampling bottle, and further processed by high-performance liquid chromatography (HPLC)^62^ using an SHIMADZU C18 column (length 250 mm, inner diameter 4.6 mm, particle size 5 μm). Mobile phase A used 0.1% acetic acid, and mobile phase B used acetonitrile. The settings were as follows: column temperature, 25 °C; wavelength, 220 nm; flow rate, 0.800 mL/min; and injection volume, 10 μL. Six types of cannabinoids were measured (mg/L): CBD, CBDV, CBDA, THC, THCV, and CBN. Samples were analyzed in triplicate. Cannabinoid content was expressed as a mass percentage, and calculations followed published procedures.^61^ All cannabinoid standards (Ceriliant, USA) were purchased from Sigma Aldrich (St. Louis, USA).

#### Metabolite extraction and liquid chromatography-mass spectrometry

Metabolite extraction, quantification, and identification from 16 flower samples were conducted by Shanghai Luming Biotechnology (Shanghai, China) according to previous methods.^63^ Each sample (approximately 200 mg) was freeze-dried. The sample was spiked with a 20 μL internal standard solution of -2-chlorophenylalanine (0.06 mg/mL in methanol), along with small steel beads. Samples were then ground (60 Hz, 2 min), subjected to ultrasonic extraction in an ice-water bath for 20 min, followed by a 20-min standing period, and centrifuged (12500 rpm, 10 min). After filtration, the supernatant was transferred to an LC sampling vial and stored at −80°C until needed for the LC-MS-based untargeted metabolomic analysis. Metabolic profiles were determined in a Dionex Ultimate 3000 RS UHPLC system, equipped with a Q-Exactive plus quadrupole-Orbitrap mass spectrometer and a heated electrospray ionization source (Thermo Fisher Scientific, Waltham, MA, USA), utilizing both positive and negative ion modes.

Metabolites from the original LC-MS data were identified and quantified in Progenesis QI version 3.0 (Nonlinear Dynamics, Newcastle, UK). Compounds were identified based on mass-to-charge ratios (M/z), secondary fragments, and isotope distributions using the Human Metabolome Database (HMDB), LipidMaps (version 2.3), Metlin, the BGI HRAM-PMDB (PMDB), and a self-built database. A data matrix was constructed and imported into R (version 3.2.0) for PCA. Orthogonal partial least squares-discriminant analysis (OPLS-DA) and partial least squares-discriminant analysis (PLS-DA) were applied to discern variations in metabolites among different groups. To prevent overfitting, model quality was assessed using 7-fold cross-validation along with 200 response permutation tests. Variable importance in projection (VIP) values from OPLS-DA were used for ranking each variable’s contribution to group discrimination. Sample categories were predicted with PLS-DA, and their relationship with metabolite expression was established. Between-group differences in metabolites were assessed with two-tailed Student’s *t*-tests. Metabolites with VIP >1.0 and *p* ≤ 0.05 were deemed to be differentially accumulated metabolites (DAMs). The KEGG database was utilized for pathway enrichment analysis of these DAMs. The KEGG IDs of the DAMs and the hypergeometric test results were used to obtain the significantly enriched pathways. The *p*-value threshold was set at ≤0.05. The smaller the values, the more significant the difference in the pathway.

#### RNA extraction, sequencing, and DEG analysis

Total RNA extraction and sequencing of six samples (CF-1, CF-2, CF-3, XF-1, XF-2, and XF-3; CF = female flower and XF = male flower) were performed using an RNAprep Pure Plant Plus Kit (DP441, Tiangen Biotech, China). Libraries were established from extracted RNA using the VAHTS Universal V5 RNA-Seq Library Preparation Kit (Vazyme, China), followed by transcriptome analysis. All these procedures were carried out by OE Biotechnology (Shanghai, China) using an Illumina HiSeq 2500 sequencer. The 150 bp paired-end raw reads were cleaned and aligned against the reference genome CS10^64^ in HISAT 2.^65^ Read counts per gene were determined using HTSeq.^66^ Biological duplication and pairing difference analysis of samples were conducted on the DESeq2^67^ V1.22.2. The read counts of each gene across all samples were first normalized (with baseMean values employed for expression-level estimation), fold changes were computed, and differential significance was tested via the negative-binomial (NB) Wald test. The q-value for each gene was derived by subjecting its *p*-value to Benjamini-Hochberg FDR correction. Differentially expressed genes were those with q-values ≤0.05 and |log2FoldChange| >1. Hierarchical cluster analysis of DEGs was conducted on R software (V 3.2.0). Functional analyses were performed with GO and KEGG databases.

#### Integrated metabolomics and transcriptomics analysis

Using transcriptomic and metabolomic data, samples were clustered in R through PCA and linear discriminant analysis. Additionally, Pearson’s correlation analysis was employed to validate the correlations between DEGs and DAMs. In Cytoscape (version 3.8.0; The Cytoscape Consortium, USA), a correlation network was constructed. Subsequently, functional annotation and enrichment analyses were conducted using the KEGG database, considering *p* ≤ 0.05 and correlation coefficients >0.8. Based on the one-to-one correspondence between samples, the correlation between features of the two omics was calculated, and heatmaps, matrix plots, and network relationship diagrams were generated. Pearson’s correlation analysis was used for the correlation analysis.

### Quantification and statistical analysis

Statistical analyses were performed with SPSS Statistics 26.0 (SPSS Inc., Chicago, IL) and GraphPad Prism 10. Results are expressed as mean ± SE (*n* ≥ 3). Student’s t-tests determined significance, with *p* < 0.05 set as the threshold.
