# Supplementary material for: Comparative analysis of gene expression and metabolites in female and male Cannabis sativa flowers
Source: iScience. 2026 Feb 7;29(3):114941. doi: 10.1016/j.isci.2026.114941 (PMC13080396; doi:10.1016/j.isci.2026.114941)
Supplement: Document S1. Figures S1–S4 [file mmc1.pdf]

iScience, Volume *29*

Supplemental information

Comparative analysis of gene expression and metabolites in female and male *Cannabis sativa* flowers

Yang Chen, Guochao Qi, Jing Zhang, Zhigang Dai, Canhui Deng, Chaohua Cheng, Zemao Yang, Jiquan Chen, Xiaoyu Zhang, Siyuan Zhu, Qing Tang, Mingbao Luan, and Ying Xu

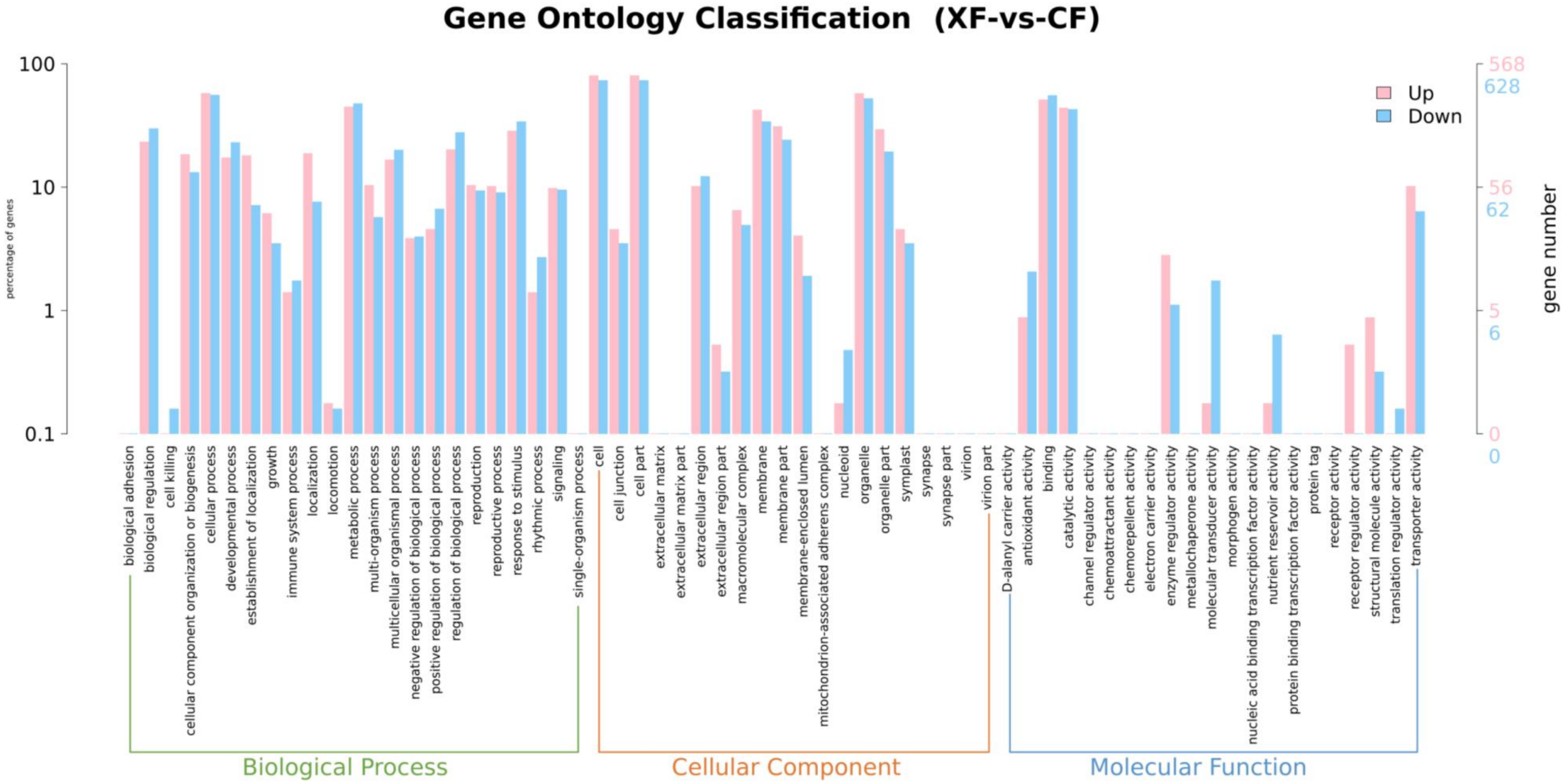

Figure S1 Gene Ontology (GO) functional enrichment analysis of male and female Cannabis sativa L. flowers

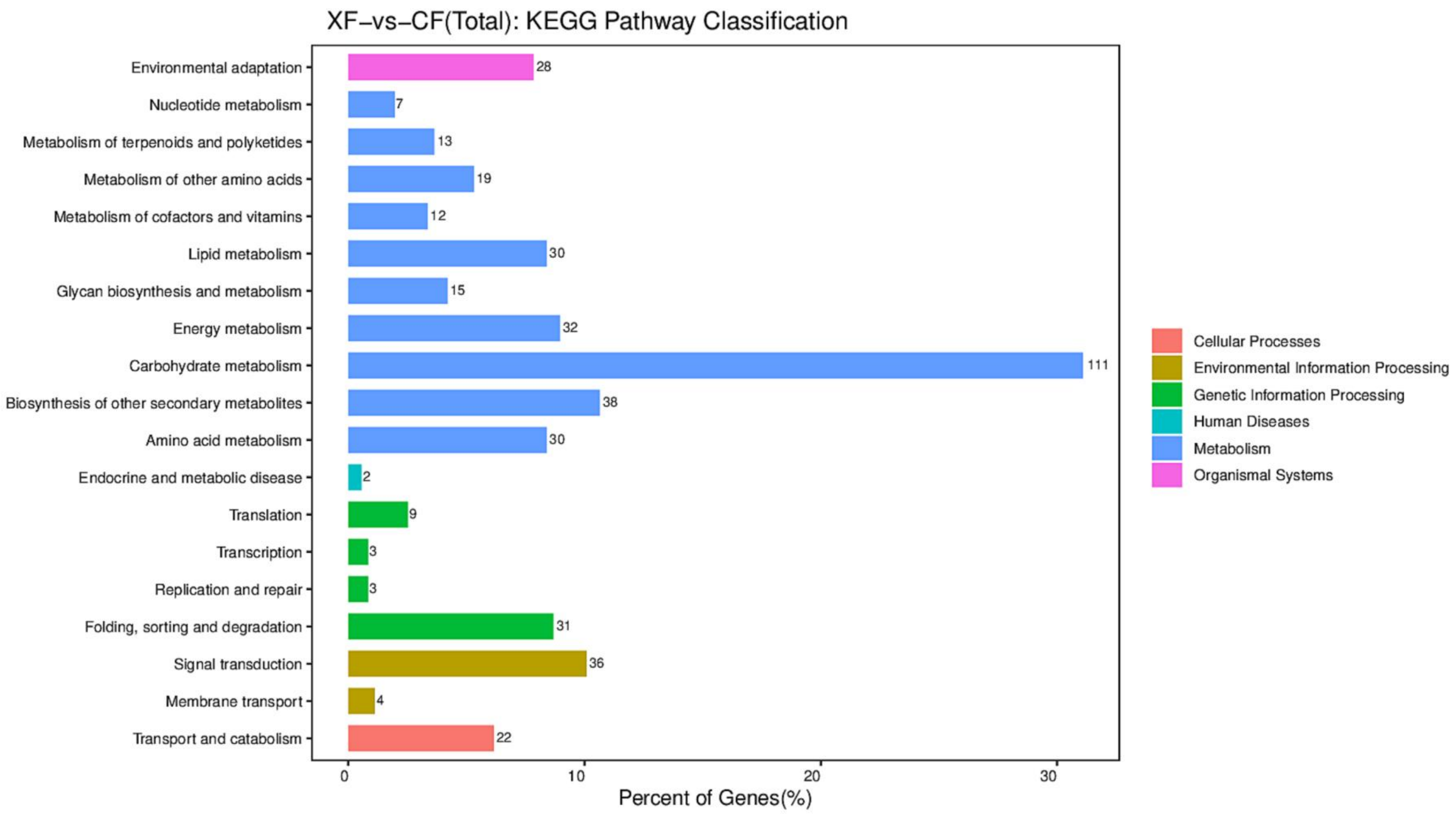

Figure S2 KEGG enrichment analysis of differentially expressed genes (DEGs) of male and female Cannabis sativa L. flowers

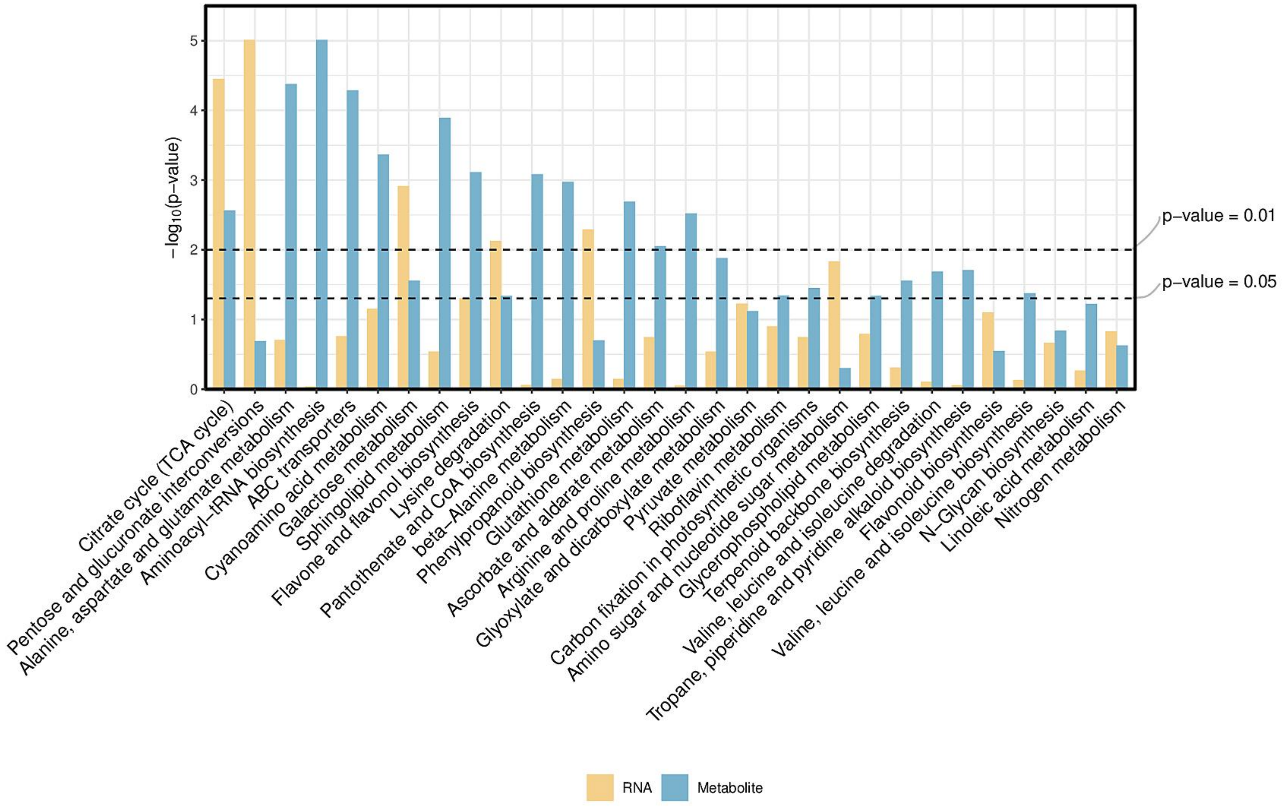

Figure S3 Bar chart of the top 30 KEGG pathways of differentially expressed genes (DEGs) and differentially accumulated metabolites (DAMs)of male and female Cannabis sativa L. flower

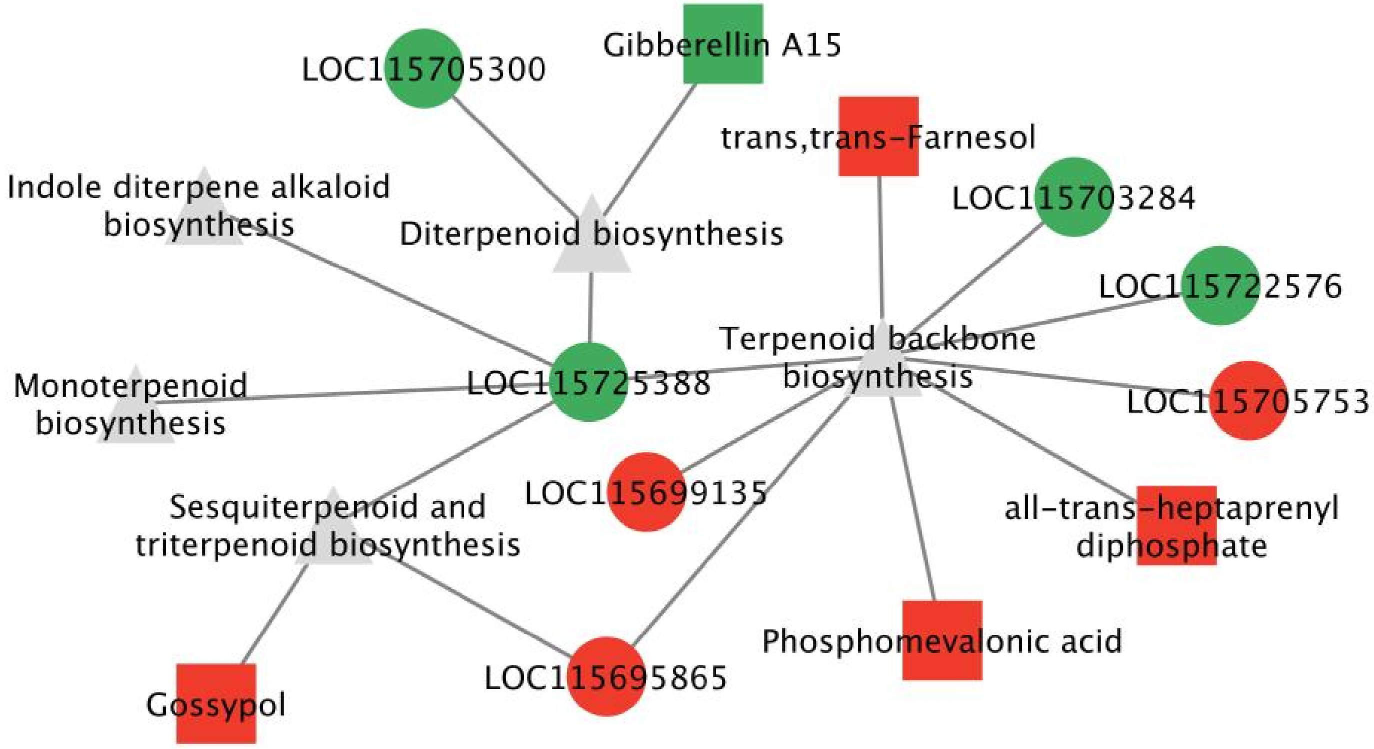

Figure S4 Comprehensive metabolomic and transcriptomic analysis of biosynthetic pathways of phenylpropanes, flavonoids and terpenes. The correlation analysis results of differentially expressed genes (DEGs) and differentially expressed metabolites (DAM) ($p<0.05$) showed that the red dots and squares represented the up-regulation of DEG and DAM, respectively, and the green dots and squares represented the down-regulation of DEG and DAM, respectivel
